# High Resolution Crystal Structures of the *Cerebratulus lacteus* Mini-Hb in the Unligated and Carbomonoxy States

**DOI:** 10.3390/ijms13078025

**Published:** 2012-06-28

**Authors:** Francesca Germani, Alessandra Pesce, Andrea Venturini, Luc Moens, Martino Bolognesi, Sylvia Dewilde, Marco Nardini

**Affiliations:** 1 Department of Biomedical Sciences, University of Antwerp, Universiteitsplein 1, B-2610 Antwerp, Belgium; E-Mails: francesca.germani@ua.ac.be (F.G.); luc.moens@ua.ac.be (L.M.); 2 Department of Physics, University of Genova, Via Dodecaneso 33, I-16146 Genova, Italy; E-Mails: pesce@fisica.unige.it (A.P.); S2950466@studenti.unige.it (A.V.); 3 Dipartimento di BioScienze, Università degli Studi di Milano, Via Celoria 26, I-20133 Milano, Italy; E-Mail: martino.bolognesi@unimi.it

**Keywords:** nerve globin, crystal structure, heme reactivity, carbon monoxide, protein matrix tunnel

## Abstract

The nerve tissue mini-hemoglobin from *Cerebratulus lacteus* (CerHb) displays an essential globin fold hosting a protein matrix tunnel held to allow traffic of small ligands to and from the heme. CerHb heme pocket hosts the distal TyrB10/GlnE7 pair, normally linked to low rates of O_2_ dissociation and ultra-high O_2_ affinity. However, CerHb affinity for O_2_ is similar to that of mammalian myoglobins, due to a dynamic equilibrium between high and low affinity states driven by the ability of ThrE11 to orient the TyrB10 OH group relative to the heme ligand. We present here the high resolution crystal structures of CerHb in the unligated and carbomonoxy states. Although CO binds to the heme with an orientation different from the O_2_ ligand, the overall binding schemes for CO and O_2_ are essentially the same, both ligands being stabilized through a network of hydrogen bonds based on TyrB10, GlnE7, and ThrE11. No dramatic protein structural changes are needed to support binding of the ligands, which can freely reach the heme distal site through the apolar tunnel. A lack of main conformational changes between the heme-unligated and -ligated states grants stability to the folded mini-Hb and is a prerequisite for fast ligand diffusion to/from the heme.

## 1. Introduction

The nerve haemoglobin (Hb) from the nemertean worm *Cerebratulus lacteus* (CerHb) is one of the smallest naturally occurring known globins, being composed of 109 amino acids instead of the ~140–160 residues typical of most globins [1,2]. Its structure shows a markedly edited globin fold with deletion of the *N*-terminal A helix, extension of the GH hinge region, and a shortened *C*-terminal H helix [3]. Both sequence and structural analyses indicate that CerHb is evolutionary equally distant from known globins, suggesting the presence of a specific mini-Hb family within the Hb superfamily. The affinity of CerHb for O_2_ is very close to that of mammalian myoglobins (Mbs), and its function has held to be the storage of O_2_ for release around axons and brain tissue during periods of burrowing under anoxic conditions [2]. From structural considerations it appears remarkable how such a small and altered globin structure can secure heme binding and the ensuing O_2_ storage function.

Despite the minimal size of the polypeptide chain, CerHb binds the heme group according to the stereochemical rules observed throughout the globin superfamily [3]. The heme Fe atom is regularly coordinated to the proximal His(93)F8 residue (amino acids have been identified with their topological site numbers as defined in the conventional globin fold), while on the (ligand binding) distal site O_2_ stabilization is achieved through a network of hydrogen bonds based on the three key residues: Tyr(11)B10, Gln(44)E7, and Thr(48)E11. Tyr(11)B10 and, to a lesser extent, Gln(44)E7 are the distal residues directly hydrogen-bonded to the O_2_ molecule, stabilizing the heme-bound form. Thr(48)E11 participates in this process by fine tuning the Tyr(11)B10 OH group orientation relative to the O_2_ ligand molecule. Indeed, structural, mutagenesis, and simulation studies indicated the presence of a dynamic equilibrium between high and low affinity states of CerHb heme distal site, driven by the ability of Thr(48)E11 to orient the Tyr(11)B10 phenolic OH group relative to the ligand in such a way that the heme-bound O_2_ would be destabilized through electrostatic repulsion of the lone electron pairs on the Tyr(11)B10 phenolic O atom [4,5]. As a result of such novel control mechanism in Hbs, based on “fractional” hydrogen bonds, the affinity of CerHb for O_2_ is only moderate (*K*_O2_ = 1 μM^−1^), and the rate of O_2_ dissociation is unexpectedly high (*k*_off_ = 200–600 s^−1^), contrary to the enhanced O_2_ affinity normally found for TyrB10-GlnE7 containing Hbs or Mbs [6].

A second distinct structural feature of CerHb is the presence of a long apolar tunnel that traverses the interior of the globin matrix from the heme pocket to the solvent, between the *C*-terminal ends of the E and H helices [3]. This tunnel allows a relatively unhindered access to the distal portion of the heme pocket and correlates with an unusually large association rate constant for O_2_ binding to CerHb (*k*′_O2_ = 240 μM^−1^·s^−1^), when compared with those of other invertebrate globins containing the Tyr-Gln active site motif (1 to 5 μM^−1^·s^−1^) [6]. Thus, loss of the A helix, which would block access to the channel, may have been an adaptive strategy to allow for rapid rates of O_2_ exchange while retaining the TyrB10-GlnE7 motif [3,4]. The structural and kinetic properties of the apolar tunnel have been extensively examined by X-ray crystallography, ligand binding kinetics, and molecular dynamics on wild type CerHb and mutants at selected sites within the tunnel (Ala(55)E18 and Leu(86)G12) [7,8]. All such data provide unambiguous experimental proof that diatomic ligands can enter and exit CerHb through the protein matrix tunnel, in preference to the more direct and “classic” E7 gate, typically found in Mb [9]. Some degree of structural heterogeneity has been highlighted at the E7 site, where both the addition of Xenon to the CerHb Leu(86)G12→Ala mutant and oxidation of wild type CerHb heme iron cause the appearance of an *out* Gln(44)E7 conformer, in which the amide side chain points to the solvent and appears to lower the barrier for ligand escape through the E7 gate. However, the observed kinetics suggest little entry and escape (<25%) through the E7 pathway, presumably because the *in* conformer, displaying an “in” conformation for residue Gln(44)E7, is thermodynamically favoured [8].

In the work presented here, we used X-ray crystallography to determine the high resolution structures of CerHb in the (ferrous) unligated and in the CO-bound states (1.9 Å and 1.5 Å resolution, respectively), and compared these to the oxygenated form of the protein previously reported. We show that the overall protein structure in the distal site and in the apolar tunnel is unperturbed by ligand binding; however, the CO and O_2_ ligands bind the heme Fe-atom with different orientations, while exploiting very similar H-bonding stabilization schemes within the distal site.

## 2. Results and Discussion

### 2.1. Overall Structures of CerHb in the Unligated and Carbomonoxy States

**Figure 1:**
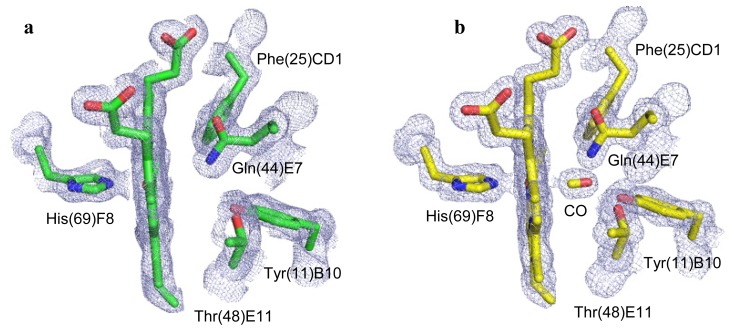
A view of the heme cavity in (**a**) unligated-CerHb, and (**b**) CerHb-CO, including distal and proximal sites, and the 2*Fo-Fc* electron density (blue mesh, contoured at 1σ level) calculated at the end of the refinement process. Residues lining the heme pocket are shown in ball-and-stick representation (unligated-CerHb in green and CerHb-CO in yellow, respectively). The heme is seen edge on; the distal cavity is on the right of the heme.

The structure of the ferrous unligated form of CerHb (unligated-CerHb) was refined to a general *R*-factor value of 18.3% (*R*-free 22.1%) for data in the 25.0–1.9 Å resolution range, with ideal stereochemical parameters (root mean square deviation, r.m.s.d.) from ideal values for bond lengths 0.014 Å, and for bond angles 1.3°) [10]. The final model, restrained refined with isotropic *B*-factors, contains 819 protein atoms, corresponding to residues Met(0) to Leu(109)H20, one heme prostetic group, 115 ordered solvent atoms (nine in two alternate positions), one glycerol molecule, two sulfate and one acetate anions (Table 1): Asp(9)B8 side-chains has been refined in a double side-chain conformation. Analysis of the electron density map at the distal iron side confirmed the absence of any ligand bound to the heme-Fe^2+^ atom (Figure 1a).

Restrained refinement of the carbomonoxy-form of CerHb (CerHb-CO) converged to a final *R*-factor value of 14.6% (*R*-free 17.9%) for data in the 24.7–1.5 Å resolution range, with ideal stereochemical parameters (r.m.s. deviation from ideal values for bond length 0.010 Å and for bond angles 1.3°) [10]. The final model contains 836 protein atoms, corresponding to residues Met(0) to Leu(109)H20, one heme prostetic group, 123 ordered solvent atoms, one glycerol molecule, two sulfate and one acetate anions (Table 1). Residues Met0, Asp(9)B8, Ser(76)G2, Ser(91)G17, and Leu(109)H20 were refined with double side-chain conformations. Additionally, four water molecules and one sulphate ion were modelled in two alternate positions. *B*-factors were refined anisotropically. Analysis of the electron density map at the distal iron side clearly indicates the presence of a diatomic ligand bound to the heme-Fe^2+^ atom (Figure 1b).

**Figure 2:**
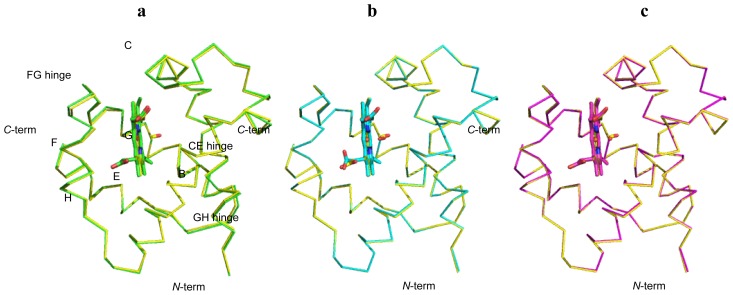
(**a**) Superimposition of unligated-CerHb (green) on CerHb-CO (yellow). All helical regions are labeled according to the globin fold topology, (**b**) Superimposition of CerHb-O_2_ (cyan) on CerHb-CO (yellow), and (**c**) CerHb-H_2_O (magenta) on CerHb-CO (yellow).

When compared to each other, the unligated-CerHb and CerHb-CO tertiary structures are very similar in their backbone, displaying a r.m.s deviation value of 0.29 Å, calculated over 109 C_α_ atom pairs (Figure 2). Furthermore, both structures conform well to those previously reported in the literature [3,8] (see below). In particular, the CerHb-CO backbone is nearly identical to those of other ligated CerHb structures, such as oxygenated CerHb (CerHb-O_2_), and aquo-met CerHb (CerHb-H_2_O), with r.m.s deviation values over 109 C_α_ atom pairs of only 0.10 Å and 0.16 Å, respectively. On the contrary, the unligated-CerHb structure superimposed onto CerHb-O_2_ and CerHb-H_2_O yielded r.m.s deviation values (over 109 C_α_ atom pairs) of 0.30 Å and 0.31 Å, respectively. Thus, despite a very good overall structural conservation, the 3D structures of CerHb in its bound states appear to cluster together based on the r.m.s.d. values.

In general, the structural deviations between CerHb unligated and ligated state structures can be ascribed to a contained breathing mode of the protein, whose tertiary structure shrinks slightly upon ligand binding. Indeed, if the solvent-excluded volume (*i.e.* the volume inside the excluded protein surface, determined using a spherical probe of 1.4 Å for a water molecule) is calculated for each of the CerHb structures, the unligated-CerHb molecule displays a 1.5%–3% larger volume than CerHb in the ligated states. At the level of secondary structure, the only significant differences are localized at the *C*-terminus of the F helix and of the FG-hinge (residues 69–73), where the coordinate displacement of the unligated-CerHb C_α_ backbone relative to CerHb-CO is 0.45 Å, and at the GH-hinge (residues 90–97), with a coordinate displacement of 0.47 Å. The net result is a small shift of the F helix away from the heme in the unligated structure, with the length of the Fe–His(69)F8 NE2 bond increasing from 2.06 Å in CerHb-CO to 2.21 Å in unligated-CerHb, while maintaining the proximal His azimuthal angle essentially unperturbed. For reference, in the CerHb-O_2_ and CerHb-H_2_O structures the Fe–His(69)F8 NE2 bond length is 2.06 Å and 2.05 Å, respectively. In the CerHb-O_2_ structure the heme group shifts slightly toward the outer rim of the globin pocket (0.29 Å) relative to its position in unligated-CerHb, and in CerHb-O_2_ or CerHb-H_2_O, however no significant differences are present for the iron position in the heme plane, with the iron atom only 0.08 Å out-of-plane at the proximal side in the unligated-CerHb.

### 2.2. CO Binding at the CerHb Distal Site

**Figure 3:**
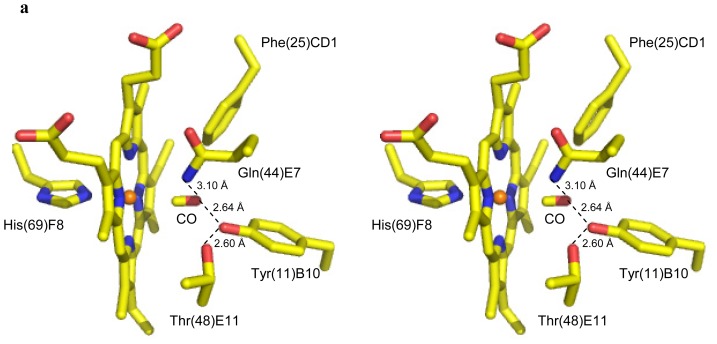

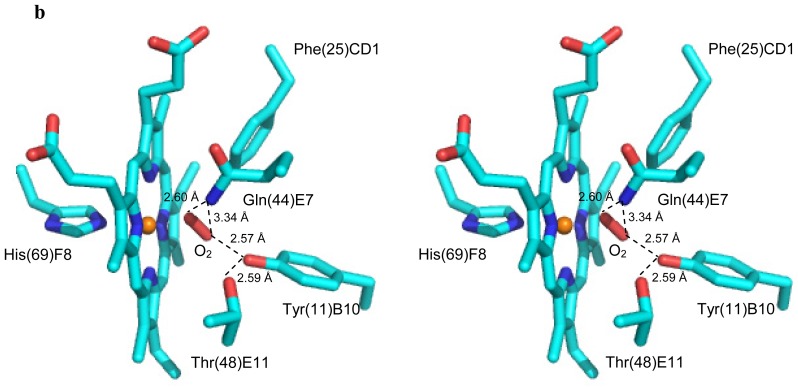
Ligand stabilization at the heme distal site of CerHb. Stereo view of the distal site region in (**a**) CerHb-CO (yellow) and (**b**) CerHb-O_2_ (cyan). The heme iron atom is shown in orange. Hydrogen bonds are drawn as dashed lines.

The electron density clearly shows a diatomic ligand coordinated to the sixth coordination site of the heme Fe-atom in CerHb-CO (Figure 1b). On the basis of the shape of the electron density, and in view of the way crystals were prepared (see below), we identified and refined it as the exogenous CO molecule. The final refined model indicates an Fe–CO distance of 2.09 Å and an Fe–C–O angle of 172° for CerHb-CO (Table 2 and Figure 3a), comparable to that reported for Mb-CO [11,12]. Two main hydrogen bonds stabilize the heme Fe^2+^-bound carbon monoxide molecule. On the one hand, the carbon monoxide O atom is linked to the Tyr(11)B10 OH group (2.64 Å), on the other, it is hydrogen bonded to the Gln(44)E7 NE2 atom (3.10 Å). Additional polar interactions contribute to the overall structural organization of the heme distal site. Specifically, the Tyr(11)B10 OH group is connected to the Thr(48)E11 OG1 atom by a strong hydrogen bond (2.60 Å), while weaker interactions connect the Tyr(11)B10 OH group to the Gln(44)E7 NE2 atom (3.06 Å), and the Gln(44)E7 NE2 atom to the Thr(48)E11 OG1 atom (3.56 Å). Moreover, the close contact (3.43 Å) occurring between the heme Fe-bound CO molecule (through its O atom) and the rim of Phe(25)CD1 should be noted, indicative of an aromatic-electrostatic contact, which further contributes to stabilization of the ligand (Figure 3a). As a result of the interlacing of side chains and hydrogen bonds, the heme Fe-bound carbon monoxide molecule is fully buried in the distal site, being totally inaccessible to solvent. If we compare the binding mode of the diatomic ligands in CerHb-CO and CerHb-O_2_ we notice that, despite the different binding geometry of the dioxygen molecule, the H-bonding network stabilizing the ligand is essentially the same in CerHb-CO and CerHb-O_2_ (Table 2 and Figure 3b).

**Figure 4:**
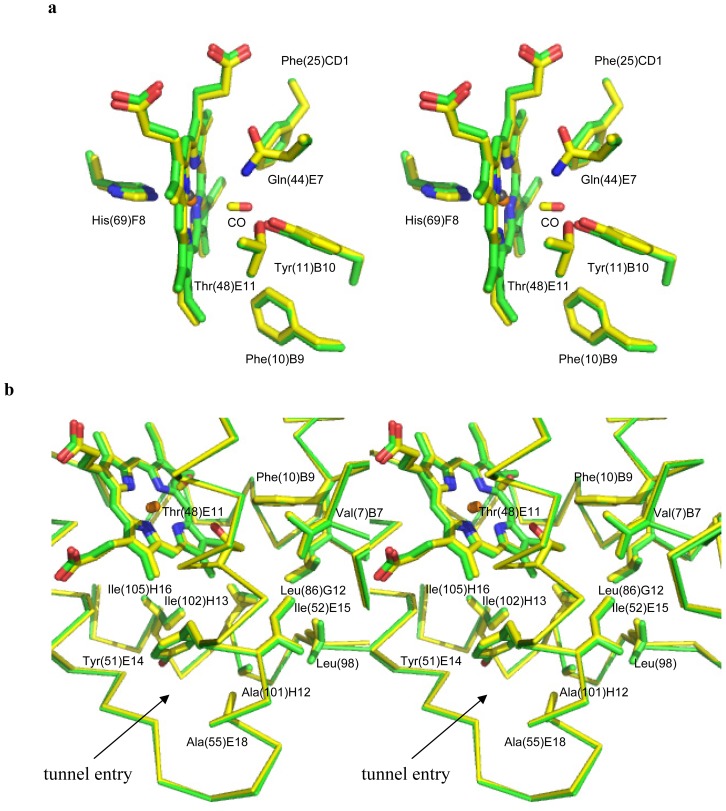
Stereo view of the structural superimposition of CerHb-CO (yellow) and unligated-CerHb (green) (**a**) at the distal site, and (**b**) at the apolar tunnel region. Relevant residues have been shown in stick representation.

The distal site residues in the CerHb-CO and unligated-CerHb structures superimpose almost perfectly, not only in their C_α_ positions but also in their side-chains, with only few exceptions: in the presence of the ligand the Tyr(11)B10 side chain rotates about 4° away from the centre of the distal site, while Phe(10)B9 move slightly in by a rotation of about 9° around the C_α_–C_β_ bond. At the same time the heme plane rotates about 4° around the CHB-CHD axis, with an inwards shift of about 0.3 Å (Figure 4a). Thus, we can conclude that ligand binding alters very marginally the structure of the distal site relative to the unbound state, and that the H-bonding network responsible for the stabilization of the bound ligand is substantially conserved independently of the nature of the diatomic ligand (either CO or O_2_).

### 2.3. Ligand Diffusion Pathway through the CerHb Apolar Tunnel

Ligand access to the heme takes place in CerHb through a wide hour-glass-shaped protein matrix tunnel connecting the distal site to a surface cleft located between the E and H helices [3,7,8]. The tunnel is lined with small hydrophobic residues, such as Tyr(51)E14, Ile(52)E15, Ala(55)E18, Leu(86)G12, Leu(98), Ala(101)H12, Ile(102)H13, and Ile(105)H16, and has a diameter that varies between 6.9 and 5.5 Å in the narrowest part, which is close to residue Leu(86)G12. The tunnel end at the heme distal site is surrounded by the side chains of Val(7)B6, Phe(10)B9, and Thr(48)E11.

The structural comparison of unligated-CerHb and CerHb-CO 3D structures indicates that ligand binding does not induce any relevant conformational change in the tunnel, whose lining residues match almost perfectly (Figure 4b). Comparable results, relative to the tunnel residues, are found when unligated-CerHb and CerHb-O_2_ 3D structures are overlayed. Such findings are particularly relevant since the tunnel lining residues are all involved in determining the free energy profile for ligand entry, as calculated using dioxygen as a probe: (*i*) free energy barrier of ~5.5 kcal/mol due to steric restrictions posed by Val(7)B6, Phe(10)B9, Tyr(11)B10, and Thr(48)E11; (*ii*) small (≤1 kcal/mol) steric barrier near Leu(86)G12; and, (*iii*) a barrier of ~2 kcal/mol near Ala(55)E18 at the tunnel exit to solvent. Furthermore, mutational studies showed that increasing the side chain size at Leu(86)G12 and Ala(55)E18 increases the extent of CO geminate recombination (to ~50%), and decreases both the association rate *k*′_O2_ and dissociation rate *k*_O2_ with little change in affinity [7,8]. Based on our data we can conclude that no perturbation of the apolar tunnel occurs as a result of exogenous ligand binding (CO or O_2_); the tunnel therefore maintains identical structure and, likely, dynamic properties, independently of the ligation state of the protein.

## 3. Experimental Section

### 3.1. Crystallization of CerHb and Derivative Preparation

CerHb was expressed and purified as described previously using a synthetic gene with codon usage optimized for expression in *Escherichia coli* [3,13]. The oxygenated derivative of CerHb (CerHb-O_2_) was crystallized by vapor diffusion techniques (protein concentration 27 mg/mL) under conditions matching those reported in the literature [3,13]. Elongated prismatic crystals (about 0.05 × 0.05 × 0.3 mm^3^) grew within 1 week. The unbound form of CerHb (unligated-CerHb) was prepared by soaking the CerHb-O_2_ crystals in a stabilizing-cryo solution containing 2.8 M ammonium sulfate, 50 mM sodium acetate, pH 5.5, 15% glycerol and 50 mM dithionite, for 30 min in a sealed well. Unligated-CerHb crystals were then quickly frozen in liquid nitrogen for data collection, or transferred in a sealed well containing the same stabilizing-cryo solution but saturated with CO for preparation of the CO-bound derivative (CerHb-CO). After 30 min, the CerHb-CO crystals were quickly frozen in liquid nitrogen for data collection.

### 3.2. Data Collection, Phasing and Refinement

High resolution data (1.9 Å for unligated-CerHb, and 1.5 Å for CerHb-CO) were collected at 100 K at the European Synchrotron Radiation Facility, Grenoble, France (beam line ID23-1). These crystals belong to the orthorombic space group *P*2_1_2_1_2_1_ (Table 1). All collected data were reduced and scaled using MOSFLM and SCALA [14,15], and phased by molecular replacement methods with the program MOLREP [16] by using the CerHb-O_2_ structure as the starting model (PDB accession code 1KR7) [3]. The crystallographic refinement was performed using the program REFMAC [17], and the program COOT [18] was used for model building/inspection. The relevant data collection and refinement statistics are reported in Table 1. The program Procheck [10] was used to assess the stereochemical quality of the protein structures and the program 3V to calculate the solvent-excluded volume of the protein structures [19]. Atomic coordinates and structure factors of unligated-CerHb and CerHb-CO have been deposited with PDB accession codes 4AVE, and 4AVD, respectively.

## 4. Conclusions

The present study investigated the structural changes that occur in CerHb upon exogenous ligand binding. The crystal structure of CerHb-CO revealed that the diatomic ligand binds to the heme Fe^2+^ atom with a coordination bond length of 2.09 Å in a linear orientation (Fe–C–O angle of 172°), markedly different if compared to the CerHb-O_2_ complex, where the diatomic ligand is coordinated to the heme Fe^2+^ atom at 1.94 Å and adopts a bent orientation, forming a 103° Fe–O1–O2 angle in the direction of the methinic CHD atom, thus pointing to the rear end of the heme [3]. Despite the different orientations, the overall binding scheme of the bound ligands is essentially the same, and stabilized through a network of hydrogen bonds based on Tyr(11)B10, Gln(44)E7, and Thr(48)E11. Tyr(11)B10 and, to a lesser extent, Gln(44)E7 are the distal residues directly hydrogen-bonded to the ligand, stabilizing the heme-bound form. Thr(48)E11 participates in this process by fine tuning the Tyr(11)B10 OH group orientation relative to the ligand molecule. Interestingly, comparison of the CerHb unligated species with CerHb-CO and CerHb-O_2_ reveals that binding of a ligand to the protein does not induce any dramatic conformational changes in the tertiary or in the secondary structures. As a result, both the distal site and the apolar tunnel, which provides access to the distal site from the solvent, are almost identical in structure in unligated-CerHb and in CerHb-CO/O_2_. Thus, CerHb represents an example of structural divergence within the globin superfamily where a minimal fold is able to bind stably the heme group and to take up and store O_2_ when the worm is in seawater, and then release it to nerve tissue when the organism burrows into mud flats and becomes anoxic [2]. To do so CerHb adapted its tertiary structure and heme distal site to achieve a Mb-like O_2_ affinity (*k*′_O2_ = 17 μM^−1^·s^−1^, *k*_O2_ = 15 s^−1^, *K*_O2_ = 1.1 μM^−1^ for Mb), but larger rates for O_2_ uptake and release (*k*′_O2_ = 240 μM^−1^·s^−1^, *k*_O2_ = 180 s^−1^, *K*_O2_ = 1.3 μM^−1^ for CerHb) [4]. Moderate O_2_ affinity stems from the presence of a Thr residue at the E11 position (E11 is an apolar amino acid in most other animal globins) that tunes the equilibrium between high and low affinity states of CerHb heme distal site by orienting the Tyr(11)B10 phenolic OH group away from the ligand [4,5]. Conversely, the need for rapid rates of O_2_ exchange during transient periods of hypoxia appears to have led to the creation of a large apolar tunnel between the E and H helices coupled with the concomitant loss of the A helix [7,8]. Thus, the Mb-like O_2_ affinity of CerHb is not merely due to a similar O_2_ binding mode, but to a more complex structural adaptation. In the case of CO binding, available data show that the main difference in the binding process can be ascribed to CO association to the heme, which is about 50-fold faster in CerHb [4]. Such a property may be related to the different ligand access paths identified in the two globins (CerHb tunnel *vs*. Mb E7-gate). As a result, also the *M* ratio between the affinity constants for CO *vs.* O_2_ is about 20-fold higher in CerHb.

Our data show that no additional structural changes are needed to promote ligand binding, which can freely reach, through the tunnel, the distal site; here, the Tyr(11)B10, Gln(44)E7, and Thr(48)E11 residues provide the H-bonding network needed for ligand stabilization.

The essentially unperturbed structure of CerHb in its different ligation states (but also in the absence of ligands) lends itself to few conclusive considerations. On one hand, such structural conservation may be expected for such a small protein, since conformational changes induced by ligand binding may turn into lower stability of the folded protein, or loss of heme affinity. On the other hand, a constant structure for the protein matrix tunnel is a prerequisite for efficient (fast) ligand diffusion to the heme, but also for its release. Both processes would become definitely slower if gated by conformational changes of residues limiting tunnel access/transit. Finally, and in consideration of the relatively simple architecture of CerHb, which under any aspect is an oxygen-binding globin, a molecular tunnel devoid of functional conformational transitions can be much more easily coded during evolution.

## Figures and Tables

**Table 1:** Data collection and refinement statistics for unligated-CerHb and CerHb-CO.

	unligated-CerHb	CerHb-CO
**Data Collection Parameters:**		
Space group	*P*2_1_2_1_2_1_	*P*2_1_2_1_2_1_
Cell dimensions (Å)	*a* = 42.1	*a* = 42.7
	*b =* 44.3	*b =* 43.5
	*c* = 62.1	*c* = 60.1
Resolution (Å)	25.4–1.9 (2.00–1.90)	30.5–1.5 (1.58–1.50)
Observations	55,282	129,825
Unique reflections	9,482	18,465
Completeness (%)	98.6 (100) a	99.6 (100.0)
*R*-merge b (%)	20.0 (35.7)	8.2 (31.9)
*I*/σ(*I*)	6.0 (4.2)	13.4 (5.7)
Multiplicity	5.8 (6.4)	7.0 (7.3)
**Refinement:**		
*R*-factor c/*R*-free (%)	18.3/22.1	14.6/17.9
Protein atoms in the a.u. d	819	836
Heme prostetic group	1	1
Carbon monoxide molecule	-	1
Water molecules	115	123
Sulfate ions	2	2
Acetate ions	1	1
Glycerol	1	1
**Model quality:**		
Overall *B*-factor (Å^2^):		
Protein and heme	13.9	13.3
Carbon monoxide molecule	-	18.9
Ions and glycerol	37.9	23.8
Water molecules	25.8	27.1
Rmsd from ideal values:		
bond lengths (Å)	0.014	0.010
bond angles (°)	1.3	1.3
Ramachandran plot (%) e:		
most favored regions	96.8	96.8
additional allowed regions	3.2	3.2

a Outer shell statistics are shown within parentheses;

b *R*-merge = ∑*_h_*∑*_i_* |*I_hi_* − <*I_h_*>|/∑*_h_*∑*_i_* *I_hi_*

c *R*-factor = ∑*_h_* ||*F_obs_*| − |*F_calc_*||/∑*_h_* |*F_obs_*| where *F_obs_* and *F_calc_* are the observed and calculated structure factor amplitudes, respectively;

d asymmetric unit;

e data produced using the program PROCHECK [10].

**Table 2:** Distances between polar atoms in the distal pockets of unligated-CerHb, CerHb-CO, and CerHb-O_2_ (covalent and hydrogen bonds are indicated as solid and dashed lines, respectively).

	unligated-CerHb	CerHb-CO	CerHb-O_2_
Fe–CO (Å)		2.09	
Fe–O_2_ (Å)			1.94
Fe–C–O angle (°)		172	
Fe–O1–O2 angle (°)			103
Tyr(11)B10 OH---O (Å)		2.64	
Tyr(11)B10 OH---O2 (Å)			2.57
Gln(44)E7 NE2---O (Å)		3.10	
Gln(44)E7 NE2---O1 (Å)			2.60
Gln(44)E7 NE2---O2 (Å)			3.34
Tyr(11)B10 OH---Thr(48)E11 OG1 (Å)	2.76	2.60	2.59
Tyr(11)B10 OH---Gln(44)E7 NE2 (Å)	3.13	3.06	3.24
Gln(44)E7 NE2---Thr(48)E11 OG1 (Å)	3.70	3.56	3.53

## References

[1] Bolognesi M., Bordo D., Rizzi M., Tarricone C., Ascenzi P. (1997). Nonvertebrate hemoglobins: structural bases for reactivity. Prog. Biophys. Mol. Biol.

[2] Vandergon T.L., Riggs C.K., Gorr T.A., Colacino J.M., Riggs A.F. (1998). The mini-hemoglobins in neural and body wall tissue of the nemertean worm, *Cerebratulus lacteus*. J. Biol. Chem.

[3] Pesce A., Nardini M., Dewilde S., Geuens E., Yamauchi K., Ascenzi P., Riggs A.F., Moens L., Bolognesi M. (2002). The 109 residue nerve tissue minihemoglobin from *Cerebratulus lacteus* highlights striking structural plasticity of the α-helical globin fold. Structure.

[4] Pesce A., Nardini M., Ascenzi P., Geuens E., Dewilde S., Moens L., Bolognesi M., Riggs A.F., Hale A., Deng P. (2004). ThrE11 regulates O_2_ affinity in *Cerebratulus lacteus* mini-hemoglobin. J. Biol. Chem.

[5] Martí M.A., Bikiel D.E., Crespo A., Nardini M., Bolognesi M., Estrin D.A. (2006). Two distinct heme distal site states define *Cerebratulus lacteus* mini-hemoglobin oxygen affinity. Proteins Struct. Funct. Bioinforma.

[6] Draghi F., Miele A.E., Travaglini-Allocatelli C., Vallone B., Brunori M., Gibson Q.H., Olson J.S. (2002). Controlling ligand binding in myoglobin by mutagenesis. J. Biol. Chem.

[7] Salter M.D., Nienhaus K., Nienhaus G.U., Dewilde S., Moens L., Pesce A., Nardini M., Bolognesi M., Olson J.S. (2008). The apolar channel in *Cerebratulus lacteus* hemoglobin is the route for O_2_ entry and exit. J. Biol. Chem.

[8] Pesce A., Nardini M., Dewilde S., Capece L., Martí M.A., Congia S., Salter M.D., Blouin G.C., Estrin D.A., Ascenzi P. (2011). Ligand migration in the apolar tunnel of *Cerebratulus lacteus* mini-hemoglobin. J. Biol. Chem.

[9] Srajer V., Ren Z., Teng T.Y., Schmidt M., Ursby T., Bourgeois D., Pradervand C., Schildkamp W., Wulff M., Moffat K. (2001). Protein conformational relaxation and ligand migration in myoglobin: a nanosecond to millisecond molecular movie from time-resolved Laue X-ray diffraction. Biochemistry.

[10] Laskowski R.A., MacArthur M.W., Moss D.S., Thornton J.M. (1993). *PROCHECK*: a program to check the stereochemical quality of protein structures. J. Appl. Crystallogr.

[11] Kachalova G.S., Popov A.N., Bartunik H.D. (1999). A steric mechanism for inhibition of CO binding to heme proteins. Science.

[12] Vojtechovsky J., Chu K., Berendzen J., Sweet R.M., Schlichting I. (1999). Crystal structures of myoglobin-ligand complexes at near-atomic resolution. Biophys. J.

[13] Pesce A., Nardini M., Dewilde S., Ascenzi P., Riggs A.F., Yamauchi K., Geuens E., Moens L., Bolognesi M. (2001). Crystallization and preliminary X-ray analysis of neural haemoglobin from the nemertean worm *Cerebratulus lacteus*. Acta Crystallogr. D Biol. Crystallogr.

[14] Leslie A.G. (2006). The integration of macromolecular diffraction data. Acta Crystallogr. D Biol. Crystallogr.

[15] Evans P. (2006). Scaling and assessment of data quality. Acta Crystallogr. D Biol. Crystallogr.

[16] Vagin A., Teplyakov A. (1997). Molecular replacement with MOLREP. Acta Crystallogr. D Biol. Crystallogr.

[17] Murshudov G.N., Vagin A.A., Dodson E.J. (1997). Refinement of macromolecular structures by the maximum-likelihood method. Acta Crystallogr. D Biol. Crystallogr.

[18] Emsley P., Cowtan K. (2004). Coot: model-building tools for molecular graphics. Acta Crystallogr. D Biol. Crystallogr.

[19] Voss N.R., Gerstein M. (2010). 3V: cavity, channel and cleft volume calculator and extractor. Nucl. Acid. Res.

